# Changes in plasma endocannabinoids concentrations correlate with 18F-FDG PET/MR uptake in brown adipocytes in humans

**DOI:** 10.3389/fmolb.2023.1073683

**Published:** 2023-07-26

**Authors:** Katarzyna Maliszewska, Katarzyna Miniewska, Adrian Godlewski, Wioleta Gosk, Malgorzata Mojsak, Adam Kretowski, Michal Ciborowski

**Affiliations:** ^1^ Department of Endocrinology, Diabetology and Internal Medicine, Medical University of Bialystok, Bialystok, Poland; ^2^ Clinical Research Centre, Medical University of Bialystok, Bialystok, Poland; ^3^ Independent Laboratory of Molecular Imaging, Medical University of Bialystok, Bialystok, Poland

**Keywords:** brown adipose tissue, endocannabinoids, humans, PET/MR, LC-QQQ-MS

## Abstract

**Introduction:** Recent data suggest a possible role of endocannabinoids in the regulation of brown adipose tissue (BAT) activity. Those findings indicate potential treatment options for obesity. The aim of this study was to evaluate the relationship between plasma endocannabinoids concentrations and the presence of BAT in humans.

**Methods:** The study group consisted of 25 subjects divided into two groups: BAT positive BAT(+), (*n* = 17, median age = 25 years) and BAT negative BAT(−), (*n* = 8, median age = 28 years). BAT was estimated using 18F-FDG PET/MR after 2 h of cold exposure. The level of plasma endocannabinoids was assessed at baseline, 60 min and 120 min of cold exposure.

**Results:** In both groups, BAT(+) and BAT(−), during the cooling, we observed a decrease of the same endocannabinoids: arachidonoylethanolamide (AEA), eicosapentaenoyl ethanolamide (EPEA) and oleoyl ethanolamide (OEA) with a much more profound decline in BAT(+) subjects. Statistically significant fall of PEA (palmitoylethanolamide) and SEA (stearoylethanolamide) concentrations after 60 min (FC = 0.7, *p* = 0.007 and FC = 0.8, *p* = 0.03, respectively) and 120 min (FC = 0.81, *p* = 0.004, and FC = 0.9, *p* = 0.01, respectively) of cooling was observed only in individuals with BAT.

**Conclusion:** We noticed the profound decline of endocannabinoids concentrations in subjects with increased 18F-FDG PET/MR uptake in BAT. Identification of a new molecules related to BAT activity may create a new target for obesity treatment.

## 1 Introduction

The endocannabinoid system (ECS) employs a huge network of molecules (receptors, ligands, and enzymes) involved in the control of multiple metabolic pathways. This system was identified in the early 1990 s during investigations on the mechanisms of delta (Schulz et al., 2021)-tetrahydrocannabinol (Δ9-THC) action, the major psychoactive principle of the hemp plant Cannabis sativa (Mechoulam, 2015). The cloning of cannabinoid receptors led to the identification of endogenous molecules capable of binding and activating them, defined as “endocannabinoids” because, despite being chemically different from THC, they are still capable of recognizing its specific binding sites (Di Marzo and Fontana, 1995).

The discovery of Δ9-THC in 1964 was a milestone in the investigation of endocannabinoid-specific receptors (Gaoni and Mechoulam, 1971). The first identified was cannabinoid receptor type 1 (CB1), primarily identified in rat brains (Matsuda et al., 1990), and later in humans (Gérard et al., 1991). In 1993, a second cannabinoid receptor, cannabinoid receptor type 2 (CB2), was identified (Munro et al., 1993). Both receptors are classified as G-protein-coupled receptors (GPCR). Besides CB1 and CB2 receptors, several studies suggest the existence of additional cannabinoid receptors. One is the transient receptor potential vanilloid type 1 (TRPV1) ion channel (Hayes et al., 2000), which was found to bind some endocannabinoid ligands. Moreover, recent data point to two other GPCRs, G protein-coupled receptor 55 (GPR55) and G protein-coupled receptor 119 (GPR119), as novel potential cannabinoid receptors (Agrawal and Lynskey, 2009).

Endocannabinoids were discovered 5 years after acknowledging CB1 and CB2 receptors. Structurally, endocannabinoids are lipid mediators generated on demand in the cell membrane from membrane phospholipid precursors, which then act on cannabinoid receptors in the same or adjacent cells (Schulz et al., 2021). The best-known endocannabinoids are N-arachidonoylethanolamide (AEA) and 2-arachidonoylglycerol (2-AG). AEA, also called anandamide, is only one of a large family of related bioactive acylethanolamides, which includes both oleoyl ethanolamide (OEA), palmitoylethanolamide (PEA) and stearoyl ethanolamide (SEA) (Borrelli and Izzo, 2009).

Endocannabinoids regulate human food intake, hunger, and energy storage by acting on the central nervous system and peripheral tissues, such as adipocytes, brown fat tissue, hepatocytes, islet cells and skeletal muscles (Schulz et al., 2021). The overactivation of ECS may promote obesity and metabolic disorders, such as hypertension, hypertriglyceridemia and insulin resistance, leading to metabolic syndrome and type 2 diabetes (Simon and Cota, 2017; Moreno et al., 2021). It seems that the downregulation of the endocannabinoid system may alleviate obesity and could be used as a new tool to combat the metabolic consequences (Kunos and Tam, 2011).

In terms of potential targets to treat obesity, the relation between the ECS and brown adipose tissue (BAT) is interesting. Due to the unique protein uncoupling protein 1 (UPC1) presence in the inner membrane of their mitochondria, BAT cells dissipate energy from fatty acids and glucose intake, resulting in heat production. BAT presence increases energy expenditure, promoting a negative energy balance (Maliszewska and Kretowski, 2021) and leading to weight loss. Imaging scans from PET/CT studies indicated high CB1 receptor density in BAT but not in white adipose tissue (WAT), suggesting a mutual relationship between endocannabinoids and brown fat (Eriksson et al., 2015). Consequently, activation of BAT may influence endocannabinoid metabolism. In the present study, we measured the plasma endocannabinoids concentrations during 2 h of cold exposure in men with and without BAT detected in PET/MR scanning.

## 2 Materials and methods

### 2.1 Human study

#### 2.1.1 Screening of subjects

Before being included in the study, each participant was informed about the project and signed written informed consent to participate in the project and samples collection. During the screening visit, the medical history was obtained from the volunteers and all subjects underwent a physical examination, routine laboratory tests (hematology, TSH, creatinine, liver enzymes, Na, K, and CRP), an electrocardiogram (ECG), and an oral glucose tolerance test (OGTT). The OGTTs were performed according to World Health Organization (WHO) recommendations, with a 75-g glucose load.

#### 2.1.2 Anthropometric measurements

The body height and weight of participants were measured using a standardized method. Bodyweight, body composition, and body fat distribution measurements were assessed using DXA scanning (enCORE™, iDXA Lunar GE Healthcare). In further analyses, the following parameters were evaluated: BMI, adipose tissue mass and percentage, visceral adipose tissue mass (VAT mass), visceral adipose tissue volume (VAT volume), the visceral adipose tissue as a percentage of body weight mass (VAT BW %), the visceral adipose tissue mass as a percentage of adipose tissue (VAT AT %), the percentage of android and gynoid fat, the android fat to gynoid fat ratio (A/G ratio), free fat mass (FFM), and lean mass.

#### 2.1.3 Cold exposure and PET/MR scanning

The second visit comprised of the cold exposure procedure and the PET/MR scanning, as described in our previous study. Briefly, all second visits were performed during the autumn and winter periods. All subjects after 8 h of fasting underwent 120 min long cold exposure. To apply protocol for cooling, water perfused blankets were used. Blood samples were taken before, after 1 hour and after 2 hours of cooling (Maliszewska et al., 2022a).

After this procedure, an 18F-fludeoxyglucose (18F-FDG) injection (4 MBq/kg of body mass) was injected in peripheral vein on upper limb, and a PET/MR scan (Biograph mMR 3T, Siemens Healthcare, Erlangen, Germany) of the whole body was started. To assess the activity, volume and presence of brown fat, firstly we manually outlined the regions of interest (ROIs), corresponding to BAT. The fusion images were composed of a summed dynamic 18F-FDG PET image and magnetic resonance (MR). Further, for the image analyses we used the software Carimas, developed at the Turku PET Centre in Finland. ROIs were drawn in image planes with a defined structure of BAT and in the aortic arch in the time frame with the highest first-pass concentration of the tracer. Regional time-activity curves (TACs) were generated, and glucose uptake rate data for the regions were estimated. The influx rate constant (Ki) of 18F-FGD for BAT was determined using the Gjedde-Patlak model. A lumped constant (LC) value of 1.14 (Virtanen et al., 2001) is normally used for all adipose tissues thus it was used here as well. The glucose uptake rate was assessed as follows: plasma glucose concentration × Ki × LC^−1^. The activation of BAT was described as a glucose uptake rate higher than 2.0 µmol × (100 g^−1^) × min^−1^, which was chosen after a visual interpretation of PET images and based on the literature data on the determination of the BAT glucose uptake rate at warm conditions, where it was always lower than 1.7 µmol × (100 g^−1^) × min^−1^ (Orava et al., 2011). Individuals in which BAT was detected were matched to the BAT positive group [BAT (+)], while subjects without detectable BAT in PET/MR images were classified as BAT negative [BAT (−)].

#### 2.1.4 Study participants

All volunteers were recruited in Department of Endocrinology, Diabetology and Internal Medicine, Medical University of Bialystok, Poland. From October to April in the years 2016–2018 we performed the cold exposure interventions and PET/MR scanning in a group of 37 healthy adult individuals. BAT was detected in 17 of them, and to make the study groups as coherent as possible, BAT negative participants matched in terms of age and BMI were chosen as a control group. Finally, the group consisted of 25 healthy, non-smoking Caucasian males divided to BAT (+) (*n* = 17) and BAT (−) (*n* = 8) study groups. The subjects in this survey were without any comorbidities (e.g., hypo- or hyperthyroidism, asthma, cardiovascular disease, renal or liver failure, and any acute or chronic diseases) and were not taking any medications (e.g., beta-blockers) or dietary supplements that could have had an impact on the results. Outside and shift workers were excluded from the study as well.

### 2.2 Blood collection and plasma endocannabinoid measurements

During the cold exposure, blood samples were collected to K3 EDTA containing tubes. Anticoagulated blood was centrifuged (10 min, 1,300 × g, RT) to obtain plasma which was stored at −80°C for further analyses. On the analysis day, samples were thawed on ice and then vortex-mixing for 2 min. Firstly, from each sample, 100 μL of plasma was transferred into new Eppendorf tubes, and 2 µL of a mixture of 7 deuterated internal standards at a concentration of 200 ppb each was added. After vortex-mixing, protein precipitation and plasma metabolite extraction was performed by adding 500 μL of cold methanol (−20°C), and the samples were incubated on ice for 10 min. After that, samples were centrifuged (21,000 × g for 10 min at 4°C), and supernatants (500 μL) were transferred into new Eppendorf tubes. Afterwards, the samples were dried in RT using the vacuum concentrator (Savant SPD 2010, Thermo Fisher Scientific, Waltham, MA, United States). The residues were reconstituted in 50 μL of methanol, and after the addition of 1 μL of 400 ppm butylated hydroxytoluene, samples were vortex-mixed for 10 min and transferred into glass vials. Quality control (QC) samples were prepared by mixing an equal volume of all samples in one batch. QC samples were prepared according to the same procedure as the rest of the samples. Stock solutions of each analyte were prepared at 10 μg/mL in ethanol. Ten calibration standard mixtures of the combined standards were prepared in the range of 0.5–4,000 ng/mL in ethanol. The calibration standard mixtures were stored at −20°C until use. The preparation of an external calibration curve for all endocannabinoids in the range of 10–80000 pg/mL was done by adding 2 µL of the calibration standard mixture of a particular concentration to 98 µL of methanol. Finally, each of the ten different concentrations of solutions was prepared according to the same procedure as the other samples.

Plasma samples were randomly quantified using ultrahigh performance liquid chromatography (1,290 Infinity II, Agilent Technology, Santa Clara, CA, United States) coupled with a tandem mass spectrometer (6495 Triple Quad LC/MS, Agilent Technologies, Santa Clara, CA, USA) equipped with iFunnel technology. During the analysis, 3 μL of the sample was injected into a thermostated (25°C) chromatographic column (Zorbax RRHD Eclipse Plus C18 (2.1 mm × 50 mm, 1.8 μm) with a Zorbax Eclipse Plus C18 (2.1 mm × 5 mm, 1.8 μm) precolumn; both Agilent Technologies). The flow rate was 0.3 mL/min with solvent A (water with 0.1% formic acid) and solvent B (acetonitrile with 0.1% formic acid). The chromatographic gradient started at 10% phase B and increased to 50% over 0.35 min, followed by an increase in phase B to 60% for another 5.0 min, remaining at this solvent ratio for 1.5 min. From 6.50 min, there was a slow increase in phase B to 70%, then 90% at 10 min; this was held until 14.7 min. Next, the gradient returned to the starting conditions (in 0.01 min) and remained at 10% of phase B for 2.3 min. Analyses were performed in the positive (ESI+) and negative (ESI−) ion modes. The multiple reaction monitoring (MRM) mode was used, and the transitions for each metabolite are shown in Table 1. The capillary voltage was set to 2 kV for positive and 3.5 kV for negative ionization mode. The nozzle voltage was 2 kV. The drying gas flow rate was 15 L/min temperature at 280°C. The nebulizer gas pressure was set at 25 psi, and the sheath gas temperature was at 350°C with a flow rate of 12 L/min.

**TABLE 1:** List of MRM transitions and electrospray source conditions.

Metabolite name	Retention time (min)	MRM transition	Collision energy (eV)	Polarity
Glycerophospho-N-Eicosapentaenoyl Ethanolamine	3.39	498.1 -> 423.9	32	ESI-
		498.1 -> 152.9	34	
Glycerophospho-N-Arachidonoyl Ethanolamine	3.20	500.1 -> 425.9	28	ESI-
		500.1 -> 171.0	32	
Glycerophospho-N-Palmitoyl Ethanolamine	4.49	452.2 -> 378.2	32	ESI-
		452.2 -> 171.0	38	
Glycerophospho-N-Oleoyl Ethanolamine	5.36	478.2 -> 403.8	28	ESI-
		478.2 -> 171.0	32	
Eicosapentaenoyl Ethanolamide	6.29	346.2 -> 267.3	16	ESI+
		346.2 -> 62.1	16	
Eicosapentaenoyl Ethanolamide-d4	6.24	350.2 -> 285.3	12	ESI+
		350.2 -> 66.2	16	
Docosahexaenoyl Ethanolamide	8.28	372.2 -> 311.2	12	ESI+
		372.2 -> 62.2	12	
Docosahexaenoyl Ethanolamide-d4	8.22	376.2 -> 293.1	14	ESI+
		376.2 -> 66.2	16	
Arachidonoyl Ethanolamide	8.37	348.2 -> 287.3	12	ESI+
		348.2 -> 62.2	16	
Arachidonoyl Ethanolamide-d4	8.32	352.2 -> 203.3	16	ESI+
		352.2 -> 66.2	12	
Palmitoyl Ethanolamide	9.59	300.1 -> 283.3	16	ESI+
		300.1 -> 62.2	17	
Palmitoyl Ethanolamide-d4	9.51	304.1 -> 287.3	12	ESI+
		304.1 -> 62.2	17	
2-Arachidonoyl Glycerol	10.39	379.2 -> 287.2	12	ESI+
		379.2 -> 269.1	18	
2-Arachidonoyl Glycerol-d5	10.34	384.2 -> 287.2	16	ESI+
		384.2 -> 269.1	10	
Oleoyl Ethanolamide	10.72	326.2 -> 309.3	22	ESI+
		326.2 -> 62.2	20	
Oleoyl Ethanolamide-d4	10.65	330.2 -> 313.3	22	ESI+
		330.2 -> 66.2	22	
Stearoyl Ethanolamide	13.00	328.2 -> 311.3	18	ESI+
		328.2 -> 62.2	16	
Stearoyl Ethanolamide-d3	12.96	331.2 -> 314.3	18	ESI+
		331.2 -> 62.2	18	

The concentrations of metabolites were calculated using an external ten-point calibration curve (in the range of 10–80000 pg/mL) based on isotope-labelled internal standards. Raw spectral data were loaded into Mass Hunter Quantitative Analysis Software (10.2, Agilent, Santa Clara, CA, United States), where the peaks were integrated. At the same time, four endocannabinoids with a glycerophosphoric group were determined using the external standard addition method (in the range of 10–80000 pg/mL). Parameters obtained during the method validation are presented in Supplementary Table S1 in the Supplementary Material File.

### 2.3 Statistical analyses

Statistical analysis of the clinical, body composition and plasma endocannabinoids data consisted of the Wilcoxon signed-rank test for the comparison of paired data, the Mann–Whitney *U* test for estimation of differences between BAT (+) and BAT (−) group and Spearman’s correlation for the analysis of numerical variables. The statistical significance level was set at 0.05 for all tests. Areas under the curve (AUCs) of endocannabinoids level during cold exposure were calculated using the linear trapezoidal method. The receiver operating characteristic (ROC) curve analysis was also conducted. All above-mentioned calculations were prepared in R (version 4.0.5, https://www.R-project.org/, accessed on the 6th of April 2022). Non-parametric methods were chosen due to the small sample size.

## 3 Results

### 3.1 Basic characteristics of study group

**FIGURE 1:**
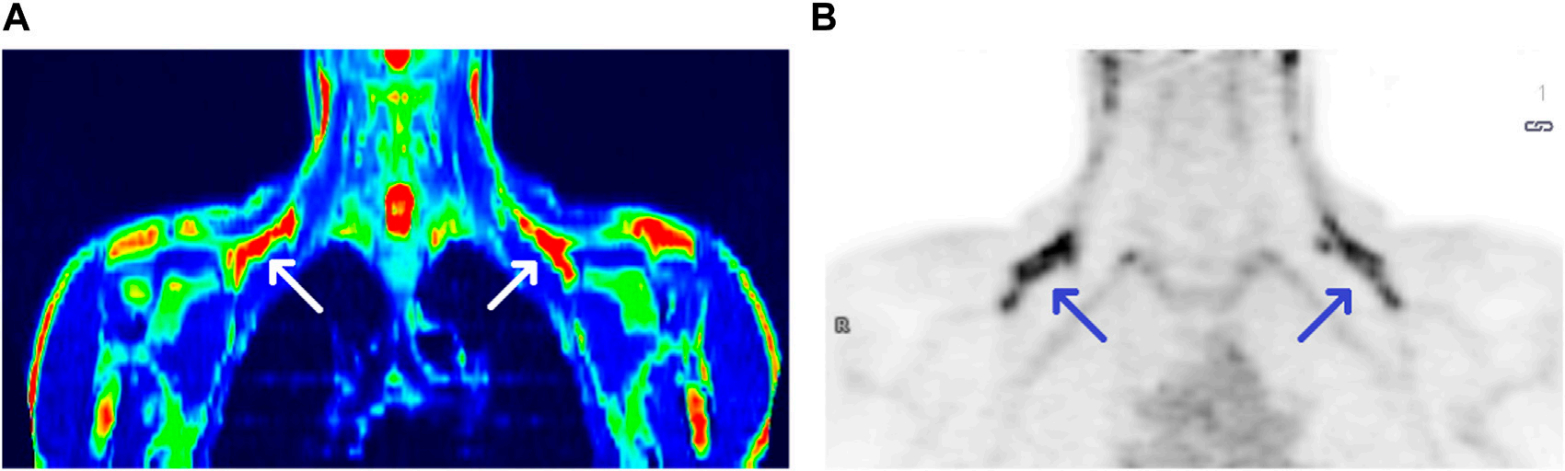
PET/MRI and PET scans of a BAT (+) individual [panel **(A,B)**, respectively]. Arrows show BAT areas in supraclavicular regions.

We observed the activation of BAT in 17 subjects, with the average activity of 3.09 µmoL × (100 g^−1^) × min^−1^ and the average volume of 27 610.94 mm^3^, both parameters calculated based on PET/MR results. Due to the fact that PET delivers rough information on metabolically active areas, in practice it is coupled with imaging techniques such as CT or MR, providing anatomical structure data. Figure 1 shows a comparison between PET/MR and PET scans (panel A and B, respectively).

The BAT (+) and BAT (−) groups were matched in terms of age, BMI, body composition, oral glucose tolerance test (OGTT) results and basic laboratory test results. Detailed characteristics are presented in Table 2.

**TABLE 2:** Characteristics of BAT (+) and BAT (−) groups. Values are shown as mean ± standard deviation.

Parameter	BAT (+) mean ± SD	BAT (−) mean ± SD	*p*-value
**N**	17	8	
BAT activity [µmol × (100 g^−1^) × min^−1^]	3.09 ± 0.70	NA	
BAT volume [mm^3^]	27 610.94 ± 42,873	NA	
Age [years]	24.88 ± 2.37	27.63 ± 3.94	0.07
fasting glucose [mg/dl]	93.35 ± 4.64	96.13 ± 5.37	0.25
AUC glucose OGTT	176.53 ± 20.7	182.63 ± 17.3	0.45
BMI [kg/m^2^]	25.71 ± 3.89	27.14 ± 3.91	0.37
AT mass [kg]	23.53 ± 14.0	23.08 ± 8.67	0.55
% AT [%]	23.28 ± 6.40	26.00 ± 5.88	0.26
VAT volume [cm^3^]	610.53 ± 547	806.75 ± 482	0.29
VAT mass [g]	576.00 ± 516	763.00 ± 452	0.24
VAT AT %	2.38 ± 1.17	3.12 ± 1.21	0.19
VAT BW %	0.60 ± 0.40	0.82 ± 0.43	0.17
android fat %	26.25 ± 9.97	30.09 ± 9.30	0.44
gynoid fat %	25.40 ± 6.38	27.80 ± 6.74	0.45
A/G ratio	1.01 ± 0.15	1.08 ± 0.18	0.31
lean mass	60.61 ± 16.37	63.73 ± 7.16	0.98
FFM	67.58 ± 7.28	66.96 ± 7.29	0.75

BAT, brown adipose tissue; BAT (+)—group possessing BAT; BAT (−)—group lacking BAT; AUC, area under the curve; OGTT, oral glucose tolerace test; BMI, body mass index; VAT, visceral adipose tissue; VAT AT %—the visceral adipose tissue as a percentage of adipose tissue mass; VAT BW %—the visceral adipose tissue mass as a percentage of body weight; A/G ratio–android fat to gynoid fat ratio; FFM, fat free mass.

### 3.2 Changes in endocannabinoids concentrations

During 2 hours of cold exposure, we noticed differences in endocannabinoid concentrations, depending on 18F-FDG uptake in brown fat depots. The percentage of change and *p*-value of the Wilcoxon signed-rank test for each metabolite are presented in Table 3.

**TABLE 3:** The median percentage of change and *p*-value of Wilcoxon signed-rank test for each endocannabinoid. Asterisk (*) stands for statistically significant values.

		BAT (−)		BAT (+)	
		60′vs. 0′	120′vs. 0′	60′vs. 0′	120′vs. 0′
AEA	*p*-value	0.02*	0.08	0.0001*	0.004*
	change [%]	−39.3	−15.1	−59.1	−32.9
EPEA	*p*-value	0.008*	0.55	0.0007*	0.008*
	change [%]	−28.0	−8.3	−32.0	−22.8
OEA	*p*-value	0.02*	0.38	0.002*	0.01*
	change [%]	−28.6	−13.3	−35.1	−24.8
PEA	*p*-value	0.06	0.31	0.008*	0.005*
	change [%]	−22.3	−15.9	−39.7	−29.9
SEA	*p*-value	0.06	0.25	0.03*	0.02*
	change [%]	−21.8	−10.3	−21.7	−32.3
2-AG	*p*-value	0.84	0.55	0.61	0.71
	change [%]	2.3	12.0	−16.0	0.9
DEA	*p*-value	0.25	0.95	0.03*	0.17
	change [%]	−13.0	−2.5	−29.9	−15.8
GP-AEA	*p*-value	0.02*	0.06	0.81	0.07
	change [%]	32.1	55.1	8.4	10.6
GP-EPEA	*p*-value	0.46	0.04*	0.82	0.07
	change [%]	16.0	10.9	0.1	15.3
GP-OEA	*p*-value	0.38	1	0.93	1
	change [%]	52.6	−6.4	2.9	−6.4
GP-PEA	*p*-value	0.20	0.46	0.58	0.75
	change [%]	49.4	11.4	−15.1	−4.3

**FIGURE 2:**
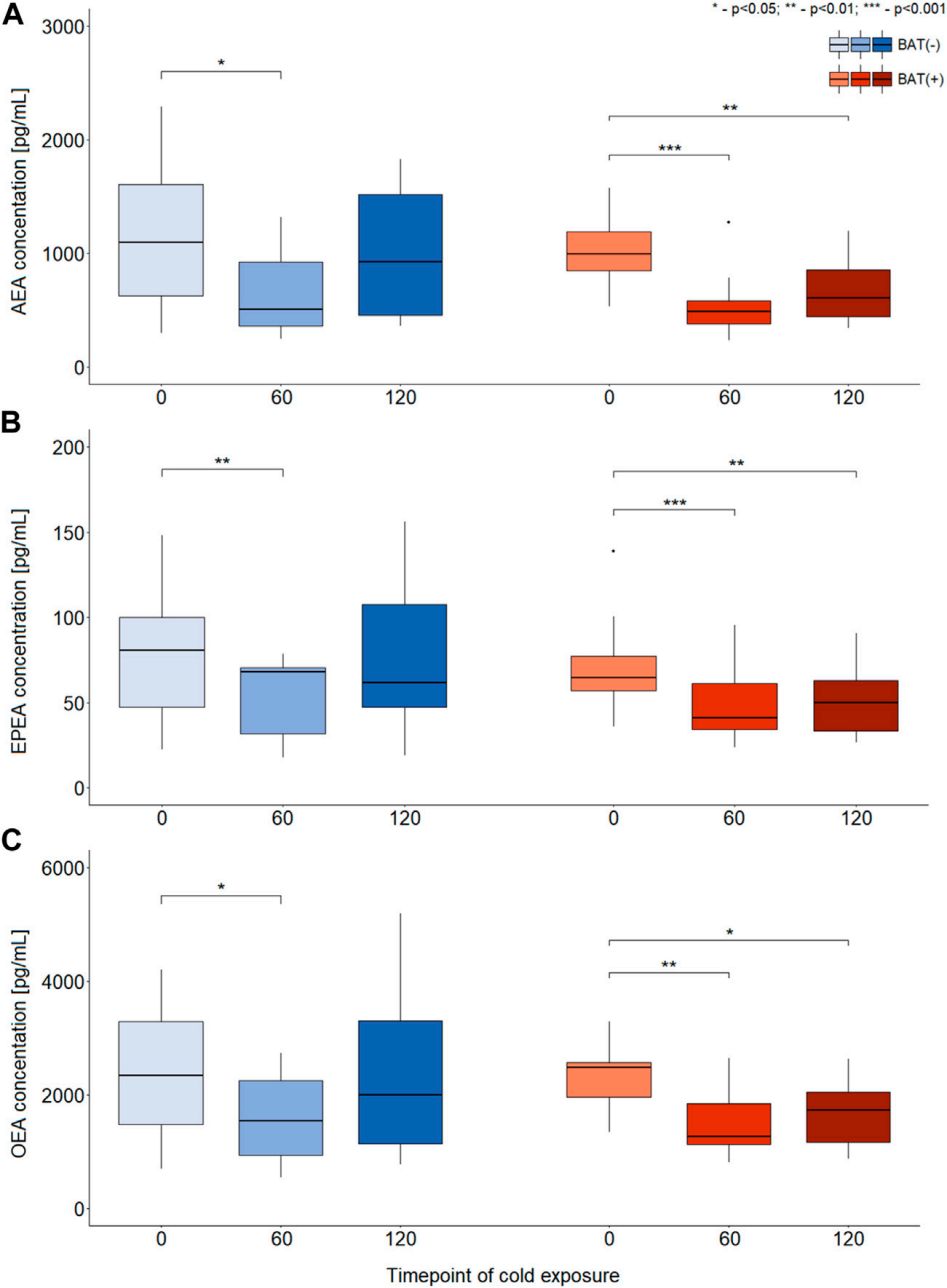
Differences in the concentration of AEA, EPEA, and OEA during CE [panels **(A–C)**, respectively]. The statistical differences were analyzed by Wilcoxon signed-rank test. Black dots stand for outliers.

In terms of AEA, EPEA, and OEA, we observed similar dynamics of changes during cold exposure in both BAT (+) and BAT (−) groups (Figures 2A–C). After 60 min of cooling, we noticed a decline in their level. In BAT (+) group, their concentrations after 120 min of cold exposure were still lower than at the beginning, whereas in BAT (−) group, their levels returned almost to their initial values.

Moreover, BAT was associated with decreased PEA and SEA concentrations after 60 and 120 min of cooling. In the BAT (−) group, the levels of these metabolites remained stable. In the BAT (−) group, after 1 h of cold exposure, an increase in glycerophospho-N-arachidonoyl ethanolamine (GP-AEA) concentration was noted. In both groups, at the end of the cooling procedure, we observed a rise in the glycerophospho-N-eicosapentaenoyl ethanolamine (GP-EPEA) level. We did not notice any statistically significant differences when the concentrations of endocannabinoids at each time of cold exposure were compared between the BAT (+) and BAT (−) groups.

**FIGURE 3:**
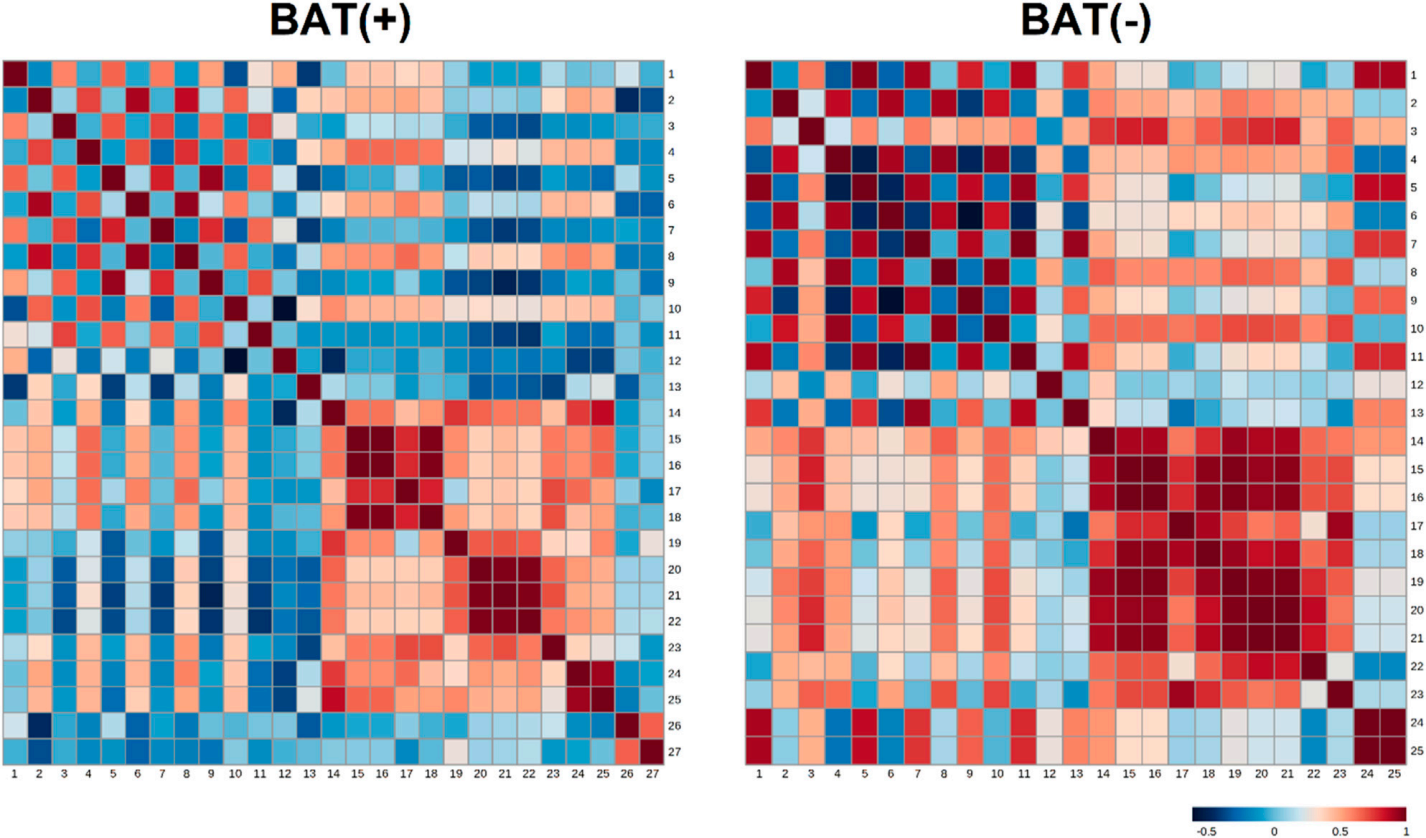
Heatmap matrix of correlations of AUC of endocannabinoids (during cold exposure) and clinical parameters, separately for BAT (−) and BAT (+) group. Each square indicates Spearman’s rank correlation coefficient which value is represented by the intensity of the colors, where red corresponds to maximal positive correlation and blue to maximal negative correlation (presented on the color scale); 1—AUC of AEA during CE; 2—AUC of GP-AEA during CE; 3—AUC of EPEA during CE; 4—AUC of GP-EPEA during CE; 5—AUC of OEA during CE; 6—AUC of GP-OEA during CE; 7—AUC of PEA during CE; 8—AUC of GP-PEA during CE; 9—AUC of SEA during CE; 10—AUC of 2-AG during CE; 11—AUC of DEA during CE; 12—fasting glucose; 13—AUC of glucose during OGTT; 14—BMI; 15—visceral adipose tissue mass; 16—visceral adipose tissue volume; 17—visceral adipose tissue mass as a percentage of adipose tissue; 18—visceral adipose tissue as a percentage of body weight; 19—adipose tissue mass; 20—adipose tissue percentage; 21—android fat percentage; 22—gynoid fat percentage; 23—android fat to gynoid fat ratio; 24—lean mass; 25—fat free mass; 26—BAT activity; 27—BAT volume.

In order to study associations between changes in endocannabinoids concentrations (represented as AUC) and clinical parameters, the analysis of correlations was performed. To better understand differences between BAT (+) and BAT (−) individuals, the analysis was performed for each group separately and correlation ratios are presented on Figure 3 as heatmaps. Additionally, the values of the correlation coefficients and respective *p*-values are available in the Data Sheet 1.xlsx Supplementary Material File.

In BAT (+) subjects, we noticed the negative correlation between android fat percentage and area under the curve (AUC) of SEA (*p* = 0.03, correlation ratio = −0,53). In the same group, the positive association was between AUC of glycerophospho-N-palmitoyl ethanolamine (GP-PEA) and lean mass (*p* = 0.01, correlation ratio = 0.58) and FFM (*p* = 0.04, correlation ratio = 0.5). Additionally, the presence of brown fat was characterized by negative correlation between AUC of GP-AEA and BAT volume (*p* = 0.049, correlation ratio = −0.49) and positive correlation between AUC of GP-AEA and lean mass (*p* = 0.04, correlation ratio = 0.5). In BAT(−) group, we observed a positive correlation between AUC-EPEA and adipose tissue mass (*p* = 0.045, correlation ratio = 0.74) and adipose tissue percentage (*p* = 0.0195, correlation ratio = 0.79). Furthermore, we assessed the relationship between the concentration of endocannabinoids in 120′ and the activity and volume of BAT, using Spearman’s correlation analysis. Although none of the association turned out to be significant (the most plausible explanation of this is the small number of observations in each group), we observed a positive tendency between the volume of BAT and the concentration of SEA (r = 0.43, *p* = 0.09).

### 3.3 ROC curves analysis

**FIGURE 4:**
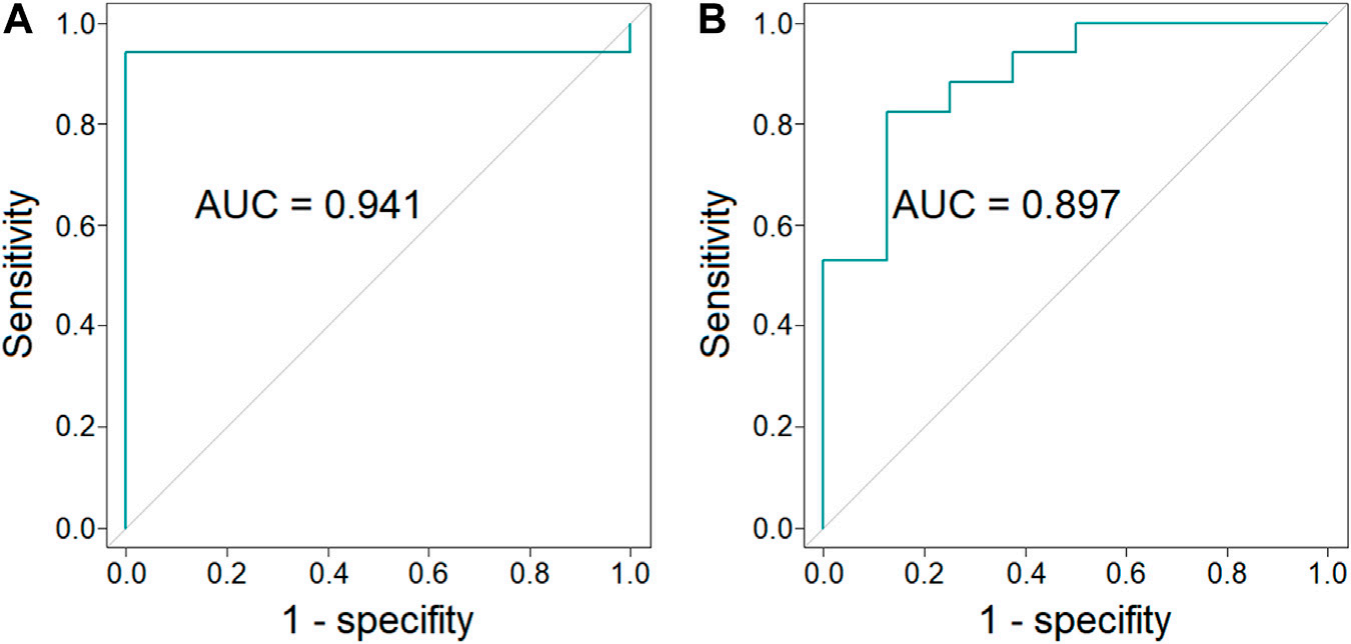
ROC curves and AUC values of the combination of the concentration of AEA, EPEA, and OEA (each in three time points) (*p*-value = 6 × 10^−5^, 95% CI = 0.826–1.000) [panel **(A)**] and the combination of the concentration of AEA and GP-AEA (each in three time points) (*p*-value = 4 × 10^−3^, 95% CI = 0.764–1.000) [panel **(B)**].

In order to evaluate a potential of endocannabinoids to serve as biomarkers indicating BAT presence, multivariate ROC curves were obtained. ROC curves were constructed for combinations of different endocannabinoids measured in different time points of cold exposure. Values used in ROC curve analysis were predicted values from logistic regression models. Odds ratio and corresponding 95% confidence intervals for models parameters are presented in Supplementary Table S2 (Supplementary Table S2). A combination of the concentration of AEA, EPEA and OEA (each in three time points) as well as a combination of concentration of AEA and GP-AEA (each in three time points) exhibited the largest value of AUC and are presented on Figures 4A, B (respectively).

## 4 Discussion

Initially, the ECS was considered to reduce pain, anxiety, motor behavior, body temperature, blood pressure, and hormone production (Crocq, 2020), but recently more evidence supports the view that endocannabinoids may regulate food intake, energy balance and body mass (André and Gonthier, 2010). Endocannabinoids act through central and peripheral mechanisms at multiple organs with adipose tissue among others (Bensaid et al., 2003; Cota et al., 2003). It has been shown that activation of BAT by cold exposure results in increase of CB1 receptors in BAT (Lahesmaa et al., 2018). Therefore, the relationship between brown adipocytes and endocannabinoids may be relevant in terms of body weight regulation. To our knowledge, the changes in endocannabinoid profile during cold exposure in men with confirmed presence of BAT have not been studied before. In the present study we observed changes in the endocannabinoid profile regardless of the presence of brown adipose tissue. However, the subjects with 18F-FDG uptake in BAT, in comparison to BAT (−) subjects, have been characterized by a significant decrease in the following endocannabinoids: AEA, EPEA, and OEA. The fall of these metabolites was also observed in BAT (−) group but was inconsiderable and returned almost to the baseline values by the end of cooling. Moreover, the activity of BAT was associated with the change of PEA and SEA, and this effect was not noticed in the BAT (−) group. Additionally, the increase of endocannabinoid precursor (GP-AEA) was observed only in the BAT (−) group after 1 h of cold exposure. Although the plasma concentration of endocannabinoids in relation to BAT presence or activity has not been studied before in humans, Lahesmaa M. et al. studied the relationships between endocannabinoid system and BAT. They quantified the density of CB1 receptor in human and rodent BAT using the PET/CT radioligand [18F]FMPEP-d2 and measured BAT activation parallel with the glucose analogue [18F]fluorodeoxyglucose (18F-FDG). As already mentioned, cold exposure markedly increased CB1 receptor density and glucose uptake in the BAT of lean men. In contrast, overweight men with reduced BAT activity exhibited decreased density of CB1 receptors in BAT, reflecting impaired endocannabinoid regulation (Lahesmaa et al., 2018). Increased CB1 receptor density in BAT may lead to accelerated uptake of endocannabinoids by this fat tissue and consequently, result in a decrease of endocannabinoids in circulation, what has been noticed in our study.

Existence of the relationship between the ECS and brown fat tissue was also suggested based on the studies with the CB1 receptor antagonist. The use of rimonabant (CB1 receptor antagonist) caused not only the appetite reduction in animals (Bensaid et al., 2003) and humans (Curioni and André, 2006), but also led to a profound increase in uncoupling protein 1 (UCP1) expression in BAT of rats accompanied by an increase of the interscapular brown adipose tissue temperature (Verty et al., 2009). Moreover, chronic administration of rimonabant to rats significantly decreased body weight with a transient reduction in food intake. Similar results were observed when mice with diet-induced obesity (DIO) were treated with peripheral CB1 receptor antagonist (BPR0912). Chronic treatment of DIO mice with BPR0912 resulted in food intake-independent weight loss with the parallel upregulation of UCP1 expression in BAT and body temperature increase (Hsiao et al., 2015). The long-term weight loss after rimonabant therapy was due to, at least in part, an elevation in energy expenditure by sympathetically denervated brown adipose tissue, mediated primarily by the central endocannabinoid system (Verty et al., 2009). Rimonabant treatment also markedly enhances insulin-mediated glucose utilization in DIO mice, independently of its anorectic and weight-reducing effects. The potent effect on insulin-stimulated BAT glucose uptake reveals a novel role for CB1 receptors as regulators of glucose metabolism (Bajzer et al., 2011). Moreover, Perwitz N et al. noticed that inhibition of peripheral CB1 receptor action in adipocytes directly promoted the transdifferentiation of white adipocytes into a mitochondria-rich, thermogenic brown fat phenotype. Increased thermogenesis and insulin sensitivity may explain a peripheral mechanism contributing to weight loss and improved glucose homeostasis in rimonabant-treated subjects (Perwitz et al., 2010). In humans, rimonabant was found effective in reducing body weight and improving the associated insulin resistance and dyslipidemias in obese/overweight people with metabolic syndrome (Després et al., 2005). It was also effective in improving glycemic control either as a monotherapy in drug-naïve diabetic patients (Rosenstock et al., 2008) or in type 2 diabetics receiving insulin (Hollander et al., 2010).

Above data from animal and human studies have demonstrated a close association between obesity and endocannabinoid system dysregulation, featured either by an overproduction of endocannabinoids or by upregulation of CB1 expression in both central and peripheral tissues (Di Marzo and Matias, 2005). The overactivation of endocannabinoid 2-AG positively correlated with visceral fat mass and indicators of insulin resistance (Blüher et al., 2006; Di Marzo et al., 2009a). The increase of plasma 2-AG and AEA levels was observed in diet induced obese mice fed with high fat diet 1 day up to 18 days. In addition, linear regression analysis on all data combined showed that body weight correlated weakly but positively with 2-AG levels and much more strongly with AEA levels (Kuipers et al., 2019). Similar to AEA, plasma concentrations of OEA, PEA, SEA were raised in response to HFD feeding. A 1-year lifestyle modification program consisting of healthy eating and regular activity/exercise induced a significant decrease in plasma endocannabinoid levels correlating with a reduction of waist circumference and biochemical metabolic risk factors in obese patients (Di Marzo et al., 2009b). In the present study we have observed (Figure 2; Table 3) that the concentrations of several endocannabinoids decrease during the first hour of cold exposure in both, BAT (−) and BAT (+) groups. However, the effect was stronger and last longer in BAT (+) group. In BAT (−) group, in the second hour of cold exposure, the concentrations of endocannabinoids were almost back to basal levels. Considering that higher concentrations of endocannabinoids are related to obesity and metabolic syndrome, their downregulation due to the activation of brown fat is probably one of the mechanisms by which BAT exert positive metabolic effects. It is worth emphasizing that OEA and PEA do not directly activate CB1 and CB2 receptors, and were suggested to act on other targets of potential importance in adipose tissue metabolism, i.e., PPARα, TRPV1 (Matias et al., 2007), and GPR55 (in the case of PEA) or GPR119 (in the case of OEA) (Godlewski et al., 2009). Therefore, the role of these two endocannabinoids in the control of BAT activation and WAT browning will have to be thoroughly evaluated when the function of their proposed receptors is explained (Krott et al., 2016). TRPV1 may be involved in energy homeostasis and the control of food intake, appetite, and energy expenditure. It may suggest that TRPV1 could be involved in the development of obesity. The mechanisms causing dysregulation have not been fully understood, but interactions with the ECS may, to some extent, explain the role of TRPV in this dysregulation (Christie et al., 2018). TRPV1 receptor could also link the endocannabinoid system with BAT. The role of the TRPV1 receptor in the process of WAT browning after treatment with capsaicin aiming to prevent diet-induced obesity in wild-type and TRPV1 (−/−) mouse models were evaluated. It was demonstrated that activation of TRPV1 channels by dietary capsaicin results in the browning of WAT, thus preventing obesity, which implies that TRPV could become a promising new target to combat obesity (Baskaran et al., 2016). In recently published paper, it was shown that the PEA in mice on a high-fat diet promoted white-to beige fat conversion and improved metabolic function of adipocytes through PPARγ (Annunziata et al., 2022). Those data suggest that interaction between endocannabinoids and its receptors is complex, especially in terms of brown and white adipose tissue and needs further evaluation.

Brown adipose tissue, based on recently published data, has been illustrated as a protective fat in terms of obesity and metabolic complications (Matsushita et al., 2014). Current knowledge cannot explain if the lack of BAT is caused by long-standing obesity or if the absence of endogenous BAT from an early age predisposes humans toward developing obesity later in life. That is why exploring the mechanism responsible for BAT activation is so interesting. In our previously published paper, we noticed that patients with BAT had been characterized with lower BMI and lower amount of visceral adipose tissue (Maliszewska et al., 2022b), which is consistent with other surveys (Orava et al., 2013; Matsushita et al., 2014; Leitner et al., 2017). Our present results indicate the negative association between endocannabinoids and android fat in BAT positive group. Based on those outcomes, we may hypothesize that maintenance of BAT activity and reduced amount of metabolic harmful visceral fat is under the condition of low concentration of cannabinoids or downregulation of the CB1 receptor. Confirmation of those conclusions needs further evaluation, but outcomes we noticed regarding the positive correlation between adipose tissue and AUC-EPEA in a group of subjects without detectable brown fat tissue may support those statements.

Except endocannabinoids, we have also quantified their intermediates, glycerophospho- (GP−) forms, which are converted to endocannabinoids by specific phosphodiesterases (Simon and Cravatt, 2006; Tsuboi et al., 2018). We observed a significant increase of GP-AEA in 1 h of cold exposure, but only in BAT (−) subjects. Additional analyses performed in this group showed a tendency (r = −0.69, *p* = 0.06) to a negative correlation between AEA and its precursor (GP-AEA). We assume that a decrease of AEA after the beginning of cooling may trigger the process of precursor formation, finally leading to an increase of AEA endocannabinoid towards baseline in 2 h of cold exposure. Those results suggest that other mechanisms, independently from brown adipocytes, could be responsible for this effect.

Our results and literature data clearly indicate the relationship between endocannabinoids and BAT presence. To further confirm this relationship, we performed a ROC analysis to evaluate if the plasma concentration of endocannabinoids may indicate BAT presence. Obtained results show that plasma concentrations of AEA, EPEA and OEA (Figure 4A) or AEA and GP-AEA (Figure 4B) can be used to indicate BAT presence.

Although obtained results extend our knowledge regarding the relationship between ECS system and BAT presence and activation, due to several limitation, should be considered preliminary. The main limitation is a relatively small number of subjects enrolled, though comparable to other studies (Orava et al., 2011; Orava et al., 2013; Blondin et al., 2017). The costs of PET/MR scanning and a tracer purchase are high, what make impossible conduction of a large-scale trial with limited university funds. Our goal was to perform the pilot study from which the outcomes should be further evaluated in a larger population and different ethnic groups. Another limitation could be the fact that we assessed the BAT glucose uptake, and it is important to note that fatty acids are the main source of energy for BAT (Ma and Foster, 1986; Maliszewska and Kretowski, 2021). Therefore, we may omit that some subjects from the BAT (+) group, defined by the glucose rate, may have a significant fatty acid uptake due to the BAT tissue. Because of the limited access to different tracers to investigate human fatty acid metabolism, we used 18F-FDG. Moreover, brown adipocytes are interspersed within white adipose tissue. Therefore, through PET detection, BAT regions could contain both BAT and some white adipocytes (Leitner et al., 2017). It is also possible that the cooling was not optimal for some of the subjects, especially those who were obese, thus resulting in false-negative results related to BAT activity.

## 5 Conclusion

Our study indicates the relationship between the ECS and BAT. Activation of brown fat was found associated with a decreased concentration of endocannabinoid metabolites. Considering that high endocannabinoids level is associated with obesity, downregulation of endocannabinoids by activated BAT could promote a metabolically favorable profile. These unique and important outcomes, observed for the first time in humans, require further research.

## Figures and Tables

**Table:** 

**18F-FDG**	[18F] fluorodeoxyglucose
**2-AG**	2-arachidonoylglycerol
**A/G ratio**	Android fat to gynoid fat ratio
**AEA**	Arachidonoylethanolamide
**AUC**	Area under the curve
**BAT**	Srown adipose tissue
**CB1**	Cannabinoid receptor type 1
**CB2**	Cannabinoid receptor type 2
**CT**	Computed tomography
**DIO**	Diet-induced obesity
**DXA**	Dual energy X-ray absorptiometry
**ECG**	Electrocardiogram
**ECS**	Endocannabinoid system
**EPEA**	Eicosapentaenoyl ethanolamide
**ESI-**	Electrospray ionization negative
**ESI+**	Electrospray ionization positive
**FC**	Fold change
**FFM**	Free fat mass
**GP-AEA**	Glycerophospho-N-arachidonoyl ethanolamine
**GPCR**	G-protein-coupled receptors
**GP-EPEA**	Glycerophospho-N-eicosapentaenoyl ethanolamine
**GP-OEA**	Glycerophospho-N-oleoyl ethanolamine
**GP-PEA**	Glycerophospho-N-palmitoyl ethanolamine
**GPR119**	G protein-coupled receptor 119
**GPR55**	G protein-coupled receptor 55
**LC**	Lumped constant
**MR**	Magnetic resonance
**MRM**	Multiple reaction monitoring
**OEA**	Oleoyl ethanolamide
**OGTT**	Oral glucose tolerance test
**PCA**	Principal component analysis
**PEA**	Palmitoylethanolamide
**PET**	Positron emission tomography
**PUFA**	Polyunsaturated fatty acids
**QC**	Quality control
**ROC**	Receiver operating characteristic curve
**ROI**	Region of interest
**SEA**	Stearoyl ethanolamide
**THC**	Tetrahydrocannabinol
**TRPV**	Transient receptor potential vanilloid
**TRPV1**	Transient receptor potential vanilloid type 1
**UPC1**	Unique protein uncoupling protein 1
**VAT AT %**	Visceral adipose tissue mass as a percentage of adipose tissue
**VAT BW %**	Visceral adipose tissue as a percentage of body weight mass
**VAT mass**	Visceral adipose tissue mass
**VAT volume**	Visceral adipose tissue volume
**WAT**	White adipose tissue
**WHO**	World Health Organization
**Δ9-THC**	Delta(9)-tetrahydrocannabinol

## Data Availability

The raw data supporting the conclusion of this article will be made available by the authors, without undue reservation.
