# Supplementary material for: Changes in plasma endocannabinoids concentrations correlate with 18F-FDG PET/MR uptake in brown adipocytes in humans
Source: Front Mol Biosci. 2023 Jul 26;10:1073683. doi: 10.3389/fmolb.2023.1073683 (PMC10411954; doi:10.3389/fmolb.2023.1073683)
Supplement: Supplementary file 1 [file DataSheet1.PDF]

Supplementary material to: **Changes in plasma endocannabinoids concentrations levels correlate with 18F-FDG PET/MR uptake in brown adipocytes in humans**

Katarzyna Maliszewska [1,*], Katarzyna Miniewska [2], Adrian Godlewski[2], Wioleta Gosk[2], Malgorzata Mojsak [3], Adam Kretowski [1], Michal Ciborowski[2]

1 Department of Endocrinology, Diabetology and Internal Medicine, Medical University of Bialystok, Poland, M.Sklodowskiej-Curie24A Bialystok, Poland;
2 Clinical Research Centre, Medical University of Bialystok, Bialystok, Poland, M.Sklodowskiej-Curie24A Bialystok, Poland;
3 Independent Laboratory of Molecular Imaging, Medical University of Bialystok, Poland, ul. Żurawia 71A 15-540 Bialystok, Poland;

* Correspondence: Katarzyna Maliszewska maliszewska.k@gmail.com;

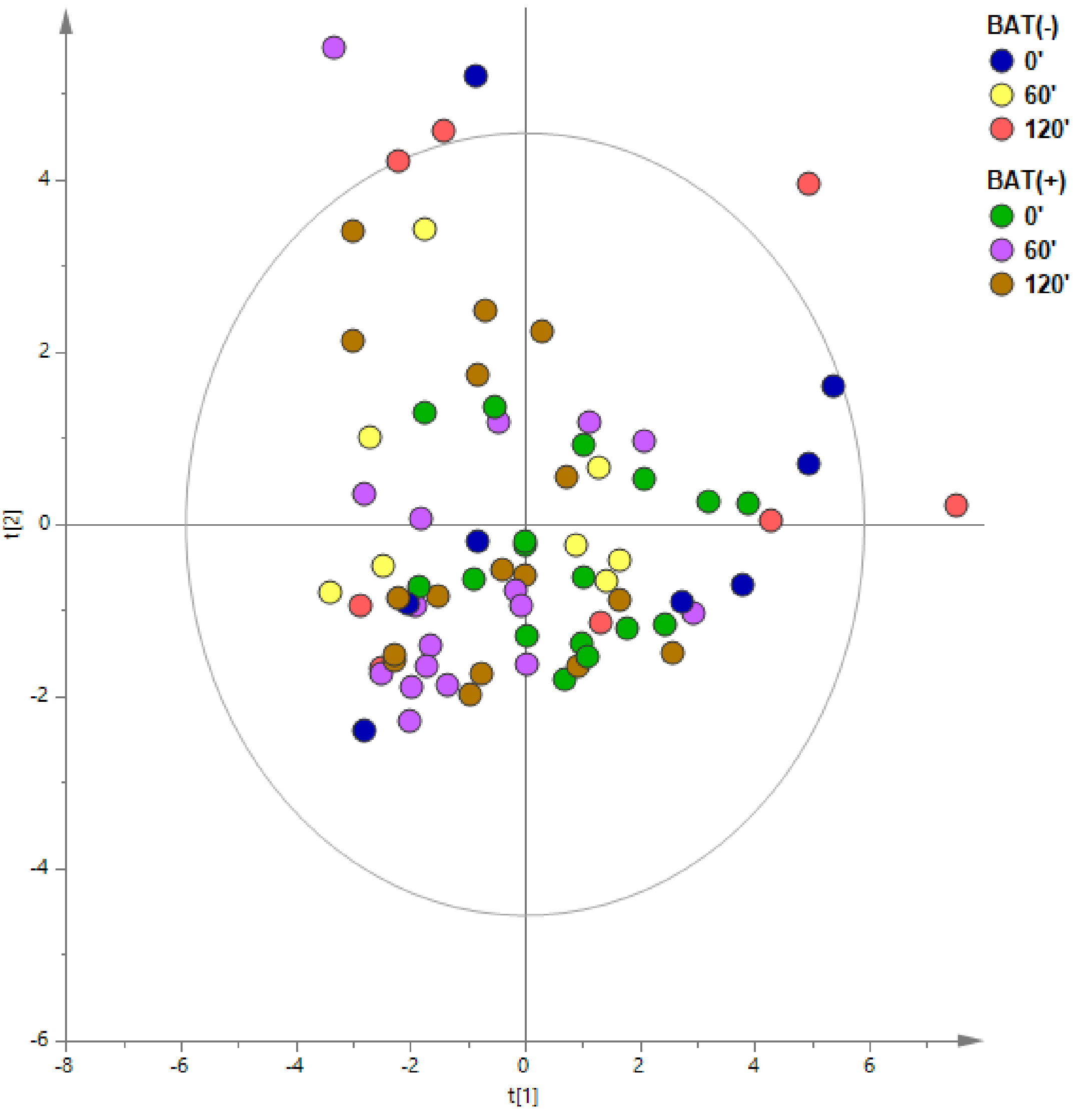

Figure S1. PCA score plots of the endocannabinoids collected from BAT(-) and BAT(+) subjects in different time points ($R^2 = 0.871$, $Q^2 = 0.651$).

Table S1. The results of validation parameters calculated for the method used in this study to measure endocannabinoids.

Metabolite	Linearity [pg/mL]	R^2	LOD [pg/mL]	LOQ [pg/mL]	Accuracy [%]	Repeatability CV [%]	Reproducibility CV [%]	Stability CV [%]	CV of QC 4000 ppt [%]	CV of QC 10 ppt [%]
GP-AEA*	400-80000	0.9972	133.33	400.00	23.85	4.74	37.08	17.67	5.21	4.75
GP-EPEA*	22-80000	0.9983	7.33	22.00	5.01	25.88	31.59	5.97	23.35	22.94
GP-PEA*	49.67-80000	0.9992	16.56	49.67	12.23	52.84	47.41	10.71	16.41	15.02
GP-OEA*	44-80000	0.9987	14.67	44.00	18.22	40.31	33.47	8.83	2.87	71.20
EPEA	21-80000	0.9982	7.00	21.00	103.80	6.77	9.17	11.72	5.37	11.99
DEA	86.67-80000	0.9989	28.89	86.67	93.18	11.73	7.36	6.33	2.41	4.50
AEA	40-80000	0.9983	13.33	40.00	93.88	6.30	28.03	7.36	1.70	5.21
PEA	49-80000	0.9977	16.33	49.00	57.44	3.31	33.33	11.01	1.58	4.41
2-AG	2000-80000	0.9962	666.67	2000.00	75.30	24.74	40.42	15.10	15.97	20.27
OEA	160-80000	0.9976	53.33	160.00	65.89	1.58	14.98	12.33	3.75	4.29
SEA	67.33-80000	0.9975	22.44	67.33	73.84	3.11	45.87	7.67	3.56	6.40

*- for these metabolites isotope-labelled internal standards were not available.

The calculated matrix effect was ranging from 0.307 to 1.349.

Table S2. Odds ratio values and 95% confidence intervals (CI) for logistic regression models used for ROC curve analysis

Parameter	Odds ratio	95% CI
Model of a combination of the concentration of AEA, EPEA and OEA (each in three time points)		
AEA 0’	1.02	0.99 – 1.06
AEA 60’	0.95	0.89 – 1.02
AEA 120’	1.02	0.97 – 1.08
EPEA 0’	0.82	0.50 – 1.15
EPEA 60’	1.54	1.01 – 2.08
EPEA 120’	0.67	0.09 – 1.24
OEA 0’	1.00	0.99 – 1.01
OEA 60’	1.01	0.99 – 1.03
OEA 120’	1.00	0.99 – 1.01
Model of a combination of concentration of AEA and GP-AEA (each in three time points)		
AEA 0’	1.0004	0.9989 – 1.009
AEA 60’	1.0001	0.9961 – 1.005
AEA 120’	0.9945	0.9889 – 1.0001
GP-AEA 0’	1.0001	0.9999 – 1.0002
GP-AEA 60’	0.9999	0.9999 – 1.0002
GP-AEA 120’	0.9999	0.9999 – 1.0001
